# Supplementary material for: Association of Polymorphisms in FSHR, INHA, ESR1, and BMP15 with Recurrent Implantation Failure
Source: Biomedicines. 2023 May 5;11(5):1374. doi: 10.3390/biomedicines11051374 (PMC10216578; doi:10.3390/biomedicines11051374)
Supplement: Supplementary file 1 [file biomedicines-11-01374-s001.zip › biomedicines-2347517-supplementary.pdf]

Table S1. Clinical variables in RIF patients stratified by *FSHR, INHA, ESR1, BMP15* polymorphisms status by ANOVA and Kruskal-Wallis tests

Genotypes	BMI (kg/m^2)	Homocysteine (µmol/L)	Folate (ng/ml)	BUN (mg/dl)	Creatinine (mg/dl)	Uric acid (mg/dl)	Total cholesterol (mg/dL)	TSH (mU/L)	E2 (pg/mL)	FSH (U/L)	LH (U/L)
	mean ± SD	mean ± SD	mean ± SD	mean ± SD	mean ± SD	mean ± SD	mean ± SD	mean ± SD	mean ± SD	mean ± SD	mean ± SD
***FSHR* rs6165 A>G**											
AA	21.0±3.3	6.7±2.1	18.4±12.7	9.8±3.1	0.8±0.1	4.1±1.1	186.5±42.4	2.3±1.6	29.4±13.9	8.9±5.5	5.2±2.5
AG	21.4±3.3	6.9±1.3	11.7±5.2	10.9±2.7	0.8±0.1	4.1±1.0	187.4±45.4	2.2±1.3	38.2±16.2	9.0±3.8	4.5±1.9
GG	20.4±2.0	8.5±1.6	9.3±4.8	11.0±2.1	0.8±0.1	3.3±0.5	169.8±29.1	2.6±1.6	30.2±13.9	8.1±2.1	4.7±0.8
P^b	0.775	0.288[a]	0.080	0.154	0.331	0.218[a]	0.592	0.809	0.007	0.508	0.457
***INHA* rs11893842 A>G**											
AA	21.6±3.6	6.5±1.4	15.0±10.3	10.3±3.0	0.8±0.1	4.2±0.9	187.7±40.8	2.6±1.9	28.6±14.5	9.2±4.3	4.8±2.4
AG	21.1±3.4	6.9±1.4	16.6±13.4	10.3±2.8	0.8±0.1	3.9±0.9	185.6±42.1	2.1±1.0	35.6±17.1	8.4±4.2	4.7±2.2
GG	20.5±2.2	7.4±2.6	13.7±6.0	10.9±3.0	0.8±0.1	4.0±1.3	181.3±47.2	2.2±1.6	35.8±11.3	9.5±5.6	5.2±1.7
P^b	0.574	0.333[a]	0.937	0.572	0.668	0.547[a]	0.516	0.742	0.014	0.542	0.313
***INHA* rs35118453 C>T**											
CC	20.9±3.0	6.9±1.5	15.4±12.5	10.7±3.1	0.8±0.1	4.1±1.0	185.4±42.6	2.4±1.7	31.2±15.1	9.2±4.4	4.8±2.3
CT	21.8±3.7	6.9±2.4	14.3±7.3	9.6±2.3	0.8±0.1	3.9±1.0	186.7±45.7	2.0±1.0	35.8±15.4	8.3±5.0	4.7±1.8
TT	19.8±1.7	6.4±1.5	18.3±6.5	11.3±2.0	0.8±0.1	3.0±0.4	171.0±11.3	2.7±1.5	44.9±15.0	9.0±1.7	6.1±1.7
P^b	0.173	0.842[a]	0.449	0.210	0.680	0.139[a]	0.750	0.541	0.067	0.095	0.190
***ESR1* rs9340799 A>G**											
AA	21.3±3.4	6.8±2.0	14.8±8.2	10.7±3.0	0.8±0.1	4.0±0.9	185.5±36.6	2.2±1.4	34.3±16.5	9.0±5.1	4.9±2.3
AG	20.6±2.6	7.4±1.0	19.3±17.4	9.8±2.6	0.8±0.1	4.1±1.4	189.0±53.0	2.4±1.6	31.9±13.5	8.9±3.7	5.0±2.1
GG	21.6±4.2	6.6±1.2	11.1±7.7	10.7±2.8	0.8±0.1	4.4±0.8	164.4±32.2	3.0±1.3	30.4±14.3	7.6±3.1	3.8±0.7
P^b	0.742	0.655[a]	0.534	0.388	0.964	0.499[a]	0.516	0.304	0.689	0.595	0.358
***ESR1* rs2234693 T>C**											
TT	21.3±4.0	6.6±1.5	13.0±6.5	10.8±2.9	0.8±0.1	4.0±0.8	190.7±41.5	2.5±1.7	30.0±12.1	8.3±4.2	4.8±2.2
TC	20.8±2.7	6.7±1.3	16.1±13.1	10.1±3.0	0.8±0.1	4.0±1.0	185.5±47.2	2.3±1.4	33.7±15.7	8.6±3.8	4.8±2.3
CC	21.6±3.1	8.0±2.7	18.4±11.6	10.5±2.5	0.8±0.1	4.3±1.5	171.7±22.8	1.9±1.1	38.5±19.2	10.7±6.4	4.9±1.4
P^b	0.605	0.108[a]	0.492	0.417	0.805	0.577[a]	0.499	0.591	0.254	0.199	0.818
***BMP15* rs3810682 C>G**											
CC	21.0±3.1	6.9±1.8	15.6±10.8	10.4±2.9	0.8±0.1	4.0±1.0	185.5±44.0	2.3±1.5	33.0±15.2	9.0±4.7	4.9±2.2
CG	22.6±4.7	7.2±1.0	8.6±1.4	11.1±2.1	0.8±0.1	3.9±1.2	181.9±14.8	2.6±0.7	36.9±18.8	7.6±1.7	4.5±0.8
GG	0.0±0.0	0.0±0.0	0.0±0.0	0.0±0.0	0.0±0.0	0.0±0.0	0.0±0.0	0.0±0.0	0.0±0.0	0.0±0.0	0.0±0.0
P^b	0.206	0.787[a]	0.310	0.338	0.258	0.842[a]	0.723	0.206	0.330	0.802	0.993
***BMP15* rs17003221 C>T**											
CC	21.2±3.3	6.9±1.8	15.5±10.8	10.4±2.9	0.8±0.1	4.0±1.0	185.4±43.1	2.3±1.5	32.7±15.2	8.8±4.6	4.8±2.2
CT	19.5±0.9	7.2±1.8	11.3±5.5	10.3±0.8	0.8±0.1	3.5±0.1	177.7±25.0	2.4±0.2	46.7±15.5	9.4±3.2	5.4±1.0
TT	0.0±0.0	0.0±0.0	0.0±0.0	0.0±0.0	0.0±0.0	0.0±0.0	0.0±0.0	0.0±0.0	0.0±0.0	0.0±0.0	0.0±0.0
P^b	0.248	0.804[a]	0.822	0.720	0.649	0.419[a]	0.944	0.406	0.062	0.384	0.254

ANOVA, analysis of variance; BMI, body mass index; BUN, blood urea nitrogen; TSH, thyroid stimulating hormone; E2, estradiol; FSH, follicle stimulationg hormone; LH, luteinizing hormone; WBC, white blood cell; Hgb, hemoglobin; PLT, platelet; PT, prothrombin time; aPTT, activated partial thromboplastin time; SD, standard deviation.

P^a values were calculated by ANOVA. P^b values were calculated by Kruskal-Wallis tests

Table S1. *Cont*

Genotypes	WBC(10^3/µL) mean ± SD	Hgb (g/dl) mean ± SD	PLT (10^3/µL) mean ± SD	PT (sec) mean ± SD	aPTT (sec) mean ± SD	CD3 (pan T) (%) mean ± SD	CD4 (helper T) (%) mean ± SD	CD8 (suppressor) (%) mean ± SD	CD19 (B cell) (%) mean ± SD	CD56 (NK cell) (%) mean ± SD
***FSHR* rs6165 A>G**										
AA	7.4±2.8	12.4±1.5	239.2±66.4	11.4±0.6	30.0±3.5	66.3±13.5	35.3±8.7	28.5±7.5	12.3±4.7	17.2±8.4
AG	7.8±3.2	12.6±1.6	240.3±63.1	11.2±0.7	29.3±3.5	68.2±8.4	34.7±8.6	29.1±8.0	11.5±4.8	18.2±9.2
GG	5.7±1.1	11.8±1.3	227.7±92.8	11.6±0.5	28.8±2.4	62.4±11.8	27.7±8.3	29.6±7.4	9.1±4.2	23.8±15.1
P^b	0.141	0.178	0.449	0.059	0.519	0.339	0.102 [a]	0.896 [a]	0.223	0.528
***INHA* rs11893842 A>G**										
AA	7.6±3.3	12.6±1.4	246.0±71.7	11.3±0.7	29.7±3.6	66.9±9.6	34.7±9.0	28.9±8.4	12.7±4.5	17.2±9.1
AG	7.6±3.1	12.3±1.5	235.0±68.8	11.3±0.6	29.0±2.8	66.7±12.9	33.4±8.9	29.8±7.8	11.1±4.8	18.0±8.9
GG	6.8±1.9	12.4±1.9	235.4±56.1	11.2±0.6	30.6±4.1	67.5±9.8	36.4±8.1	26.9±6.3	11.5±4.9	19.7±10.9
P^b	0.816	0.584	0.682	0.612	0.325	0.973	0.415 [a]	0.357 [a]	0.218	0.751
***INHA* rs35118453 C>T**										
CC	7.2±3.1	12.4±1.4	239.0±66.4	11.3±0.6	29.5±3.2	66.5±12.4	34.6±9.1	29.1±7.6	11.7±4.6	18.0±9.5
CT	7.8±2.8	12.6±1.7	242.8±69.5	11.3±0.7	29.6±3.9	68.0±9.1	33.6±8.5	29.2±8.3	12.2±5.1	17.0±8.5
TT	7.6±1.6	12.3±2.6	203.0±58.9	11.1±0.4	30.7±2.6	65.8±9.4	38.1±6.2	24.6±4.2	8.2±2.4	24.9±11.7
P^b	0.364	0.400	0.595	0.607 [a]	0.535	0.887	0.562 [a]	0.371	0.159	0.264
***ESR1* rs9340799 A>G**										
AA	7.7±3.0	12.4±1.7	244.9±71.9	11.2±0.6	29.3±3.2	69.5±9.0	35.6±8.2	29.7±7.9	12.2±5.1	16.2±8.5
AG	7.1±3.0	12.7±1.3	231.0±58.0	11.3±0.6	29.7±3.8	62.4±13.6	32.2±9.4	27.5±7.4	10.5±4.1	22.6±10.4
GG	6.5±1.9	11.8±1.3	220.0±58.5	11.8±0.6	31.6±3.2	69.5±1.3	36.8±8.1	29.8±8.1	14.0±1.4	14.5±3.0
P^b	0.496	0.270	0.432	0.010	0.118	0.019	0.166 [a]	0.417 [a]	0.062	0.002
***ESR1* rs2234693 T>C**										
TT	7.5±2.9	12.5±1.6	234.0±62.1	11.1±0.7	29.2±3.3	70.7±10.3	35.6±8.5	31.4±8.4	11.8±4.0	15.1±7.7
TC	7.2±3.0	12.5±1.4	244.9±73.3	11.4±0.6	29.7±3.6	65.3±8.4	33.2±8.8	27.3±6.8	12.1±5.4	19.7±10.5
CC	8.0±2.9	12.3±1.9	229.8±57.7	11.4±0.6	30.2±3.2	64.4±17.7	36.0±9.0	28.8±8.2	10.3±3.1	19.2±8.4
P^b	0.412	0.948	0.873	0.079	0.358	0.020	0.363 [a]	0.063 [a]	0.469	0.028
***BMP15* rs3810682 C>G**										
CC	7.5±3.0	12.5±1.5	237.2±68.6	11.3±0.6	29.7±3.4	66.8±11.4	34.4±8.7	29.0±7.7	11.3±4.4	18.4±9.4
CG	6.7±2.2	12.1±1.5	257.1±40.7	11.5±0.6	27.5±1.9	68.0±9.7	34.9±9.5	27.8±7.7	15.3±7.3	14.8±9.7
GG	0.0±0.0	0.0±0.0	0.0±0.0	0.0±0.0	0.0±0.0	0.0±0.0	0.0±0.0	0.0±0.0	0.0±0.0	0.0±0.0
P^b	0.657	0.415	0.125	0.473	0.085	0.670	0.877 [a]	0.685 [a]	0.169	0.751
***BMP15* rs17003221 C>T**										
CC	7.4±2.9	12.4±1.5	239.4±66.7	11.3±0.6	29.6±3.4	67.0±11.3	34.6±8.7	28.8±7.7	11.8±4.6	18.0±9.4
CT	8.3±3.7	12.3±3.4	219.0±81.7	10.9±0.4	27.9±4.6	64.0±1.4	26.0±7.1	32.0±8.5	4.0±0.0	22.5±9.3
TT	0.0±0.0	0.0±0.0	0.0±0.0	0.0±0.0	0.0±0.0	0.0±0.0	0.0±0.0	0.0±0.0	0.0±0.0	0.0±0.0
P^b	0.600	0.434	0.877	0.111	0.414	0.402	0.169 [a]	0.565 [a]	0.018	0.528

ANOVA, analysis of variance; BMI, body mass index; BUN, blood urea nitrogen; TSH, thyroid stimulating hormone; E2, estradiol; FSH, follicle stimulating hormone; LH, luteinizing hormone; WBC, white blood cell; Hgb, hemoglobin; PLT, platelet; PT, prothrombin time; aPTT, activated partial thromboplastin time; SD, standard deviation.

P^a values were calculated by ANOVA. P^b values were calculated by Kruskal-Wallis tests

Table S2. Clinical variables in controls stratified by *FSHR, INHA, ESR1, BMP15* polymorphisms status by ANOVA and Kruskal-Wallis tests

Genotypes	BMI (kg/m²) mean ± SD	Homocysteine (μmol/L) mean ± SD	Folate (ng/ml) mean ± SD	BUN (mg/dl) mean ± SD	Creatinine (mg/dl) mean ± SD	Uric acid (mg/dl) mean ± SD	Total cholesterol (mg/dL) mean ± SD
***FSHR* rs6165 A>G**							
AA	22.2±3.9	7.8±2.7	13.0±8.5	8.8±2.9	0.6±0.1	3.8±1.0	208.2±58.1
AG	21.7±3.2	5.2±2.2	15.1±7.0	8.8±2.7	0.6±0.2	3.9±1.0	220.5±57.4
GG	22.8±2.3	4.9±6.2	8.1±0.0	8.8±2.9	0.6±0.2	4.2±1.2	217.3±54.2
P^b	0.392	0.088[a]	0.245	0.944	0.328[a]	0.499	0.430[a]
***INHA* rs11893842 A>G**							
AA	21.3±2.7	6.1±2.8	11.6±4.1	9.1±2.9	0.6±0.2	3.8±1.0	215.9±63.3
AG	22.5±3.8	6.9±4.1	15.5±10.5	8.8±2.9	0.6±0.1	3.8±1.1	216.4±55.7
GG	22.1±3.5	6.1±1.6	14.0±5.1	8.4±2.2	0.6±0.2	4.0±1.0	211.4±52.4
P^b	0.307	0.823[a]	0.596[a]	0.528	0.558	0.489	0.800
***INHA* rs35118453 C>T**							
CC	22.1±3.0	5.8±2.6	13.6±6.9	8.7±2.8	0.6±0.1	3.8±1.1	214.8±61.4
CT	21.9±4.2	7.5±3.6	14.7±8.8	9.0±2.8	0.6±0.2	4.0±0.9	216.4±51.5
TT	0.0±0.0	5.8±0.0	0.0±0.0	8.7±1.8	0.6±0.2	4.0±1.0	214.0±34.7
P^b	0.469	0.455[a]	0.734	0.836	0.786	0.445	0.984[a]
***ESR1* rs9340799 A>G**							
AA	22.2±3.8	6.5±1.8	13.4±6.7	8.7±2.8	0.6±0.2	3.9±1.1	208.8±56.7
AG	21.5±2.6	7.2±5.2	15.8±10.1	9.0±2.9	0.6±0.2	3.8±0.9	232.6±57.7
GG	25.5±3.0	0.5±0.0	0.0±0.0	8.8±2.8	0.6±0.2	3.8±0.8	202.0±44.2
P^b	0.263	0.117[a]	0.611	0.805	0.398	0.975	0.042[a]
***ESR1* rs2234693 T>C**							
TT	22.5±4.4	6.3±1.8	12.8±5.5	8.1±2.7	0.6±0.2	4.0±1.3	212.7±54.4
TC	21.8±2.7	7.0±3.4	14.5±9.0	9.1±2.9	0.6±0.2	3.8±0.9	218.7±62.2
CC	21.4±3.1	0.5±0.0	17.7±0.0	9.6±2.5	0.6±0.2	3.8±0.7	211.5±49.4
P^b	0.775	0.110[a]	0.615	0.012	0.475[a]	0.966	0.771[a]
***BMP15* rs3810682 C>G**							
CC	22.0±3.5	6.3±3.0	14.2±7.6	8.8±2.9	0.6±0.2	3.9±1.1	216.6±58.2
CG	22.2±2.8	8.3±0.0	8.2±0.0	8.5±2.0	0.6±0.2	3.9±0.7	205.6±50.1
GG	0.0±0.0	0.0±0.0	0.0±0.0	0.0±0.0	0.0±0.0	0.0±0.0	0.0±0.0
P^b	0.770	0.525[a]	0.446[a]	0.789	0.325	0.687	0.421[a]
***BMP15* rs17003221 C>T**							
CC	22.0±3.4	6.4±3.1	14.1±7.6	8.8±2.8	0.6±0.2	3.9±1.0	215.0±57.4
CT	22.9±6.6	6.9±0.0	10.1±0.0	8.4±1.9	0.6±0.2	4.3±1.0	223.8±55.9
TT	0.0±0.0	0.0±0.0	0.0±0.0	0.0±0.0	0.0±0.0	0.0±0.0	0.0±0.0
P^b	1.000	0.876[a]	0.610[a]	0.748	0.492	0.299	0.713

ANOVA, analysis of variance; BMI, body mass index; BUN, blood urea nitrogen; TSH, thyroid stimulating hormone; E2, estradiol; FSH, follicle stimulating hormone; LH, luteinizing hormone; WBC, white blood cell; Hgb, hemoglobin; PLT, platelet; PT, prothrombin time; aPTT, activated partial thromboplastin time; SD, standard deviation.

P^a values were calculated by ANOVA. P^b values were calculated by Kruskal-Wallis tests

Table S2. *Cont*

Genotypes	TSH (mU/L) mean ± SD	E2 (pg/mL) mean ± SD	FSH (U/L) mean ± SD	LH (U/L) mean ± SD	WBC(10^3/μL) mean ± SD	Hgb (g/dl) mean ± SD	PLT (10^3/μL) mean ± SD	PT (sec) mean ± SD	aPTT (sec) mean ± SD
***FSHR* rs6165 A>G**									
AA	1.4±0.8	28.6±15.0	8.1±3.3	3.4±1.9	8.2±2.3	12.3±1.1	232.9±59.2	10.9±2.3	29.2±3.6
AG	1.6±1.0	25.0±14.7	7.4±2.1	4.0±3.8	7.9±2.0	12.3±1.2	226.0±58.8	10.6±0.9	29.3±3.5
GG	2.0±0.8	24.3±12.5	9.0±2.2	3.7±1.6	7.3±2.2	12.1±1.6	236.8±90.6	10.3±0.8	28.2±3.0
P^b	0.048	0.610	0.027	0.429	0.215 [a]	0.923	0.678 [a]	0.038	0.383 [a]
***INHA* rs11893842 A>G**									
AA	1.7±1.0	26.0±12.2	8.3±2.8	3.3±1.4	7.7±1.7	12.3±1.0	230.0±75.2	10.5±0.8	29.1±4.1
AG	1.4±0.9	25.6±13.7	7.8±3.1	3.8±3.2	8.1±2.4	12.3±1.4	232.6±62.6	10.8±2.1	29.2±3.4
GG	1.6±1.0	31.5±20.7	8.5±1.9	4.2±2.5	8.1±2.3	12.3±1.1	223.4±40.8	10.6±0.7	29.0±2.7
P^b	0.175	0.904	0.144	0.659	0.484 [a]	0.992	0.754 [a]	0.684	0.610
***INHA* rs35118453 C>T**									
CC	1.6±1.0	27.1±14.8	8.1±2.6	3.6±2.8	7.8±2.0	12.2±1.2	235.0±68.6	10.6±0.9	29.1±3.7
CT	1.6±0.9	25.2±13.5	7.9±3.4	3.9±2.0	8.0±2.5	12.4±1.3	221.5±53.9	10.9±2.5	29.1±3.2
TT	1.3±0.5	0.0±0.0	0.0±0.0	0.0±0.0	9.2±2.9	12.8±0.5	229.8±43.1	10.4±0.6	29.0±3.0
P^b	0.933	0.569	0.321	0.217	0.295 [a]	0.456	0.378 [a]	0.658	0.930
***ESR1* rs9340799 A>G**									
AA	1.5±0.9	27.0±15.3	8.2±3.1	3.9±2.9	7.8±2.3	12.4±1.2	228.8±67.7	10.6±0.9	29.2±3.5
AG	1.7±1.0	25.5±12.6	7.9±2.1	3.1±1.6	8.3±2.0	12.2±1.2	234.9±54.6	10.9±2.7	29.2±3.5
GG	1.5±0.8	29.3±0.6	5.7±0.3	2.0±0.5	7.2±2.0	12.1±1.2	219.6±39.3	10.3±0.6	27.6±3.0
P^b	0.868	0.807	0.131	0.081	0.263 [a]	0.634	0.738 [a]	0.459	0.264
***ESR1* rs2234693 T>C**									
TT	1.4±0.9	27.7±15.7	8.4±3.2	3.7±3.2	8.1±2.5	12.3±1.4	231.4±58.5	10.7±1.0	29.4±3.9
TC	1.5±1.0	27.4±14.8	7.7±2.5	3.7±2.1	8.0±2.0	12.3±1.1	232.8±69.4	10.8±2.2	29.2±3.3
CC	1.8±0.9	22.3±9.4	8.0±2.4	3.3±1.7	7.6±2.0	12.2±0.9	218.6±55.0	10.4±0.6	28.1±2.8
P^b	0.281	0.370	0.394	0.884	0.580 [a]	0.597	0.555 [a]	0.484	0.198
***BMP15* rs3810682 C>G**									
CC	1.6±0.9	27.3±14.2	8.1±2.8	3.7±2.7	7.9±2.3	12.3±1.2	230.6±61.3	10.7±1.7	29.2±3.5
CG	1.5±1.0	13.2±11.9	7.9±3.4	3.5±0.8	8.0±1.2	12.0±0.9	225.4±77.7	10.5±0.7	28.9±3.4
GG	0.0±0.0	0.0±0.0	0.0±0.0	0.0±0.0	0.0±0.0	0.0±0.0	0.0±0.0	0.0±0.0	0.0±0.0
P^b	0.827	0.030	0.588	0.542	0.852 [a]	0.062	0.724 [a]	0.643	0.605
***BMP15* rs17003221 C>T**									
CC	1.6±0.9	26.6±14.6	8.1±2.8	3.7±2.6	7.9±2.2	12.3±1.2	229.7±64.0	10.7±1.6	29.0±3.4
CT	0.4±0.2	26.7±9.1	7.6±3.6	3.7±1.6	8.9±1.6	12.4±0.9	239.3±35.7	10.5±0.6	31.2±5.4
TT	0.0±0.0	0.0±0.0	0.0±0.0	0.0±0.0	0.0±0.0	0.0±0.0	0.0±0.0	0.0±0.0	0.0±0.0
P^b	0.030	0.802	0.291	0.632	0.258 [a]	0.875	0.694 [a]	0.905	0.301

ANOVA, analysis of variance; BMI, body mass index; BUN, blood urea nitrogen; TSH, thyroid stimulating hormone; E2, estradiol; FSH, follicle stimulating hormone; LH, luteinizing hormone; WBC, white blood cell; Hgb, hemoglobin; PLT, platelet; PT, prothrombin time; aPTT, activated partial thromboplastin time; SD, standard deviation.

P^a values were calculated by ANOVA. P^b values were calculated by Kruskal-Wallis tests

Table S3. Synergic effect of FSHR, INHA, ESR1, BMP15 polymorphisms with clinical risk factor.

Variables	*FSHR* rs6165 GG		*FSHR* rs6165 AA+AG		*INHA* rs11893842 AA		*INHA* rs11893842 AG+GG		*INHA* rs35118453 CC		*INHA* rs35118453 CT+TT		*ESR1* rs9340799 AA		*ESR1* rs9340799 AG+GG	
	AOR (95% CI)	*P*	AOR (95% CI)	*P*	AOR (95% CI)	*P*	AOR (95% CI)	*P*	AOR (95% CI)	*P*	AOR (95% CI)	*P*	AOR (95% CI)	*P*	AOR (95% CI)	*P*
Homocysteine																
<7.94μmol/L	1.000 (Reference)		2.662 (1.219-5.813)	0.014	1.000 (Reference)		1.016 (0.650-1.588)	0.943	1.000 (Reference)		1.202 (0.777-1.861)	0.409	1.000 (Reference)		1.222 (0.790-1.891)	0.368
≥7.94μmol/L	20.163 (1.356-299.878)	0.029	12.638 (3.286-48.609)	0.0002	2.740 (0.369-20.321)	0.324	6.180 (1.819-20.996)	0.004	8.210 (2.163-31.165)	0.002	2.629 (0.514-13.439)	0.246	4.464 (1.412-14.113)	0.011	10.725 (1.175-97.878)	0.036
Folate																
>8.12 ng/ml	1.000 (Reference)		2.644 (1.211-5.772)	0.015	1.000 (Reference)		1.051 (0.671-1.647)	0.827	1.000 (Reference)		1.151 (0.743-1.785)	0.528	1.000 (Reference)		1.168 (0.754-1.808)	0.488
≤8.12 ng/ml	20.163 (1.356-299.878)	0.029	14.298 (3.565-57.343)	0.0002	3.992 (0.642-24.814)	0.138	8.090 (2.070-31.618)	0.003	7.104 (1.834-27.514)	0.005	5.684 (1.013-31.897)	0.048	3.892 (1.195-12.678)	0.024	0.000 (0.000-0.000)	0.000
E2																
<37.6 pg/mL	1.000 (Reference)		2.154 (0.979-4.740)	0.057	1.000 (Reference)		0.912 (0.566-1.470)	0.704	1.000 (Reference)		0.949 (0.588-1.532)	0.832	1.000 (Reference)		1.319 (0.827-2.104)	0.245
≥37.6 pg/mL	4.961 (0.651-37.820)	0.122	11.411 (4.351-29.925)	<0.0001	2.389 (0.802-7.113)	0.118	6.541 (2.913-14.684)	<0.0001	3.236 (1.555-6.734)	0.002	14.580 (4.087-52.012)	<0.0001	5.328 (2.579-11.010)	<0.0001	6.538 (2.150-19.885)	0.001
FSH																
<9.6 U/L	1.000 (Reference)		2.362 (1.070-5.213)	0.033	1.000 (Reference)		1.092 (0.677-1.764)	0.718	1.000 (Reference)		1.113 (0.704-1.760)	0.647	1.000 (Reference)		1.118 (0.706-1.770)	0.635
≥9.6 U/L	1.663 (0.278-9.958)	0.577	3.009 (1.149-7.880)	0.025	2.080 (0.774-5.587)	0.146	2.922 (1.273-6.707)	0.011	2.105 (1.043-4.248)	0.038	4.296 (1.334-13.831)	0.015	1.803 (0.873-3.725)	0.112	5.511 (1.816-16.724)	0.003
LH																
<5.12 U/L	1.000 (Reference)		1.710 (0.797-3.666)	0.168	1.000 (Reference)		1.220 (0.751-1.900)	0.422	1.000 (Reference)		1.123 (0.702-1.795)	0.630	1.000 (Reference)		1.124 (0.705-1.791)	0.625
≥5.12 U/L	0.489 (0.053-4.485)	0.527	7.658 (2.742-21.388)	0.0001	12.053 (3.145-46.187)	0.0003	4.011 (1.832-8.782)	0.001	5.462 (2.398-12.439)	0.0001	3.979 (1.588-9.974)	0.003	3.298 (1.584-6.865)	0.001	12.305 (3.358-45.084)	0.0002

AOR: adjusted by age. † Folate 8.12ng/ml were lower 25% cut-off each level in RIF patients and controls. ‡ Homocysteine 7.94 μmol/L and LH 5.12 U/L, FSH 9.6 U/L, E2 37.6pg/mL were upper 15% cut-off each level in RIF patients and controls.

Table S3. *Cont*

Variables	*ESR1* rs2234693 TT		*ESR1* rs2234693 TC+CC		*BMP15* rs17003221 CC		*BMP15* rs17003221 CT		*BMP15* rs3810682 CC		*BMP15* rs3810682 CG	
	AOR (95% CI)	*P*	AOR (95% CI)	*P*	AOR (95% CI)	*P*	AOR (95% CI)	*P*	AOR (95% CI)	*P*	AOR (95% CI)	*P*
Homocysteine												
<7.94μmol/L	1.000 (Reference)		1.289 (0.828-2.006)	0.260	1.000 (Reference)		1.401 (0.619-3.168)	0.418	1.000 (Reference)		1.590 (0.440-5.753)	0.480
≥7.94μmol/L	6.626 (1.132-38.787)	0.036	5.825 (1.656-20.487)	0.006	3.784 (0.209-68.531)	0.368	7.855 (2.074-29.741)	0.002	0.000 (0.000-0.000)	0.000	7.506 (1.474-38.215)	0.015
Folate												
>8.12 ng/ml	1.000 (Reference)		1.365 (0.873-2.134)	0.172	1.000 (Reference)		1.449 (0.642-3.266)	0.372	1.000 (Reference)		1.580 (0.436-5.720)	0.486
≤8.12 ng/ml	6.818 (1.588-29.281)	0.010	9.382 (1.816-48.482)	0.008	0.000 (0.000-0.000)	0.000	9.953 (2.422-40.906)	0.001	0.000 (0.000-0.000)	0.000	20.820 (2.298-188.672)	0.007
E2												
<37.6 pg/mL	1.000 (Reference)		1.169 (0.729-1.876)	0.518	1.000 (Reference)		2.046 (0.770-5.438)	0.151	1.000 (Reference)		1.872 (0.407-8.601)	0.420
≥37.6 pg/mL	3.459 (1.231-9.713)	0.019	6.997 (3.114-15.722)	<0.0001	0.000 (0.000-0.000)	0.000	8.870 (2.914-27.004)	0.000	13.554 (0.564-325.531)	0.108	9.475 (1.882-47.711)	0.006
FSH												
<9.6 U/L	1.000 (Reference)		1.173 (0.739-1.864)	0.499	1.000 (Reference)		1.296 (0.566-2.967)	0.540	1.000 (Reference)		1.144 (0.356-3.681)	0.822
≥9.6 U/L	1.454 (0.534-3.961)	0.464	3.371 (1.501-7.571)	0.003	0.620 (0.055-6.969)	0.698	1.725 (0.649-4.581)	0.274	0.000 (0.000-0.000)	0.000	1.419 (0.395-5.102)	0.592
LH												
<5.12 U/L	1.000 (Reference)		1.167 (0.731-1.863)	0.517	1.000 (Reference)		1.335 (0.588-3.031)	0.490	1.000 (Reference)		2.087 (0.455-9.582)	0.344
≥5.12 U/L	3.180 (1.103-9.164)	0.032	6.161 (2.718-13.966)	<0.0001	4.356 (0.228-83.360)	0.329	3.289 (1.228-8.805)	0.018	18.855 (0.540-657.939)	0.105	5.741 (1.066-30.912)	0.042

AOR: adjusted by age. † Folate 8.12ng/ml were lower 25% cut-off each level in RIF patients and controls. ‡ Homocysteine 7.94 μmol/L and LH 5.12 U/L, FSH 9.6 U/L, E2 37.6pg/mL were upper 15% cut-off each level in RIF patients and controls.
